#  The Molecular Prevalence of Viral Infections in Transplant Candidates with Bone Marrow Suppression, Shiraz, Southern Iran, 2010 

**Published:** 2013-05-01

**Authors:** B. Mohammadi, R. Yaghobi, M. Dehghani, A. Behzad Behbahani

**Affiliations:** 1 *Shiraz Transplant Research Center, Namazee Hospital, Shiraz University of Medical Sciences, Shiraz, Iran *; 2 *Hematology Research Center and Bone marrow Transplant Unit, Namazee Hospital, Shiraz University of Medical Sciences, Shiraz, Iran *; 3 *School of Paramedical Sciences, Shiraz University of Medical Sciences, Shiraz, Iran *

**Keywords:** Transient bone marrow suppression, DNA virus, RNA virus

## Abstract

Background: Transient bone marrow suppression, characterized by acute inability of the bone marrow to produce circulating blood cells, may strongly relate to the pathogenesis of some viral infections.

Objective: To study the prevalence of some DNA and RNA viruses in patients with transient bone marrow suppression.

Methods: EDTA-treated blood samples were collected from 27 patients with clinically- and laboratory-confirmed transient bone marrow suppression. The genomic DNA of hepatitis B virus, adenovirus, polyomavirus BK, and parvovirus B19, and genomic RNA of hepatitis C and G viruses were extracted and amplified by sensitive and specific in-house simple and nested PCR and RT-PCR protocols, respectively. The risk factors that might be related to the studied viral infections were analyzed.

Results: Hepatitis B virus infection was diagnosed in 9 (33%) of 27 patients; adenovirus infection in 2 (7%); and parvovirus B19 infection in 7 (26%) of 27 patients. The genomic DNA of polyomovirus BK was not detected in any patients. Both hepatitis C and G viruses were found in 3 (11%) of 27 patients.

Conclusion: Diagnosis of the high prevalence of hepatitis B virus, and parvovirus B19 in patients with transient bone marrow suppression, reflects the importance of these viral infections in introducing bone marrow suppression. This hypothesis should be confirmed in further studies.

## Introduction

Transient bone marrow suppression is characterized by acute inability of the bone marrow to produce adequate number of circulating blood cells leading to pancytopenia [1, 2]. The majority of these patients are idiopathic as their etiologies remain unexplained. Understanding the exact etiology of the transient bone marrow suppression is important for specific diagnosis and treatment [3-5]. The mechanisms underlying the bone marrow suppression may result from various hematological disorders and extra-hematological abnormalities such as malignancies, infectious diseases and nutritional deficiencies [6]. Historically, limited number of studies exists evaluating the cause of transient bone marrow suppression. Also, the previously published studies on transient cytopenia have been limited by the referral nature of patient population [7].

Viral infections are among important etiologies involved in the development of the transient bone marrow suppression [7-11]. The marrow is often cellular, but rare cases of bone marrow suppression may result from viral suppression or using antiviral drugs [8]. Human parvovirus B19 is the most common virus became suspicious of bone marrow suppression [12-14]. Association between viral hepatitis and development of bone marrow suppression was reported in some case reports [15, 16]. In some studies, the implication of viral hepatitis A-C infections in bone marrow suppression was presented controversially [15, 16]. In some cases, infectious mononucleosis is subclinical—observation of the reactive lymphocytes and positive serological results are consistent with a recent infection. Our earlier report also reflects the importance of other herpes viruses in the pathogenesis of transient marrow suppressions [17]. Therefore, in this study, for the first time, the prevalence of some DNA and RNA viruses including hepatitis B virus, adenovirus, polyomovirus BK, parvovirus B19, hepatitis C virus, and hepatitis G virus were evaluated in patients with transient bone marrow suppression.

## Patients and Methods

Twenty-seven patients with transient bone marrow suppression admitted to Namazee Hospital, Shiraz, southern Iran, who were candidates for hematopoietic stem cell transplantation, were enrolled in this cross-sectional study. The diagnosis of the bone marrow suppression was made by an expert hematologist. Patients who had at least one of the following criteria were excluded from the study. The criteria included other hematological abnormalities, hematological malignancies, infiltrating disorders, and exposure to organ phosphorus toxins and oil or drug components. 

The molecular and antigenic viral assays were performed in a double blind way to abolish any possible biases. The genomic DNA and RNA of the hepatitis B virus, hepatitis C virus, hepatitis G virus, adenovirus, polyomovirus BK, and parvovirus B19 were extracted from EDTA-treated blood samples collected from each participant, and diagnostically amplified by various sensitive and specific in-house simple and multiplex PCR and RT-PCR protocols. 

**Molecular analysis**

**Extraction of viral RNA and DNA genomes**

The genomic DNA and RNA of hepatitis B virus, adenovirus, polyomovirus BK, and parvovirus B19 were extracted from the collected plasma of EDTA-treated blood samples of the patients by DNP kit (CinnaGen, Iran) according to the manufacturer’s instruction. The genomic RNA of hepatitis C and G viruses were extracted from the plasma by RNX-Plus kit (CinnaGen, Iran) according to the manufacturer’s instructions.

**PCR and RT-PCR protocols**

**Adenovirus: **The presence of adenovirus genomic DNA was evaluated in plasma samples by an in-house qualitative PCR method standardized with confirmed adenovirus genomic DNA as positive control and a laboratory-documented virus-free negative control. The primer sequences were originally designed to amplify a fragment of the gene II of adenovirus which is highly conserved among different genotypes (Table 1). The PCR mixture contained 5 μL of 10X PCR buffer, 1 μL MgCl_2_ (50 mM), 1.5 μL dNTP (10 mM), 1 μL of each primer (20 pmol), 0.5 μL Taq (2.5 unit), and 5 μL of the sample DNA. The thermocycling condition included one cycle at 94 °C for 3 min, 35 cycles (at 94 °C for 1 min, at 62 °C for 1 min, and at 72 °C for 1 min), and finally one cycle at 72 °C for 5 min.

**Table1:** The primer sequences used for in-house PCR and RT-PCR

Viruses	Primer Sequences	Length of amplification fragment (bp)
Adenovirus-F	5'-TTC CTC TAT CTC AGA CAC TGG CTC A-3'	81
Adenovirus-R	5'-CCA AGC GGC CTC TGA TAA CCA-3'	
polyomavirus BK-F	5'-CAA GTG CCA AAA CTA CTA AT-3'	326
polyomavirus BK-R	5'-TGC ATG AAG GTT AAG CAT-3'	
Parvovirus B19-OF	5' -AGTTCTTTTCAGCTTTTA- 3'	247
Parvovirus B19-OR	5' -GTAGACACTGAGTTTACT- 3'	
Parvovirus B19-IF	5' -TATAAGTTTCCTCCAGTGCC- 3'	188
Parvovirus B19-IR	5' -GGCCCTGGCAGTAAGTTTTG- 3'	
Hepatitis C virus-OF	5'-CCC CTG TGA GGA ACT ACT GTC- 3'	225
Hepatitis C virus-OR	5'-TGC ACG GTC TAC GAG ACC TC- 3'	
Hepatitis C virus-IF	5'-CAC GCA GAA AGC GTC TAG CCA TG- 3'	
Hepatitis C virus-IR	5'-TCG CAA GCA CCC TAT CAG GCA G- 3'	
Hepatitis G virus-OF	5'-GGT CGT AAA TCC CGG TCA CC- 3'	188
Hepatitis G virus-OR	5'-CCC ACT GGT CCT TGT CAA CT- 3'	
Hepatitis G virus-IF	5'-TAG CCA CTA GAG GTG GGT CT- 3'	
Hepatitis G virus-IR	5'-ATT GAA GGG CGA CGT GGA CC-3'	

**Hepatitis B virus:** Hepatitis B virus genomic DNA was identified in patients samples using specific primers amplifying a fragment of the surface gene by a qualitative hepatitis B virus PCR detection kit (CinnaGen, Iran) according to the manufacturer’s instruction.

**Polyomavirus BK:** The genomic DNA of polyomovirus BK was diagnosed in studied patients by an in-house qualitative protocol using originally designed specific primers amplifying a fragment of *Vp1* gene (Table 1). The sensitivity and specificity of this protocol was optimized using confirmed pPolyomovirus BK genomic DNA as positive control and a laboratory-documented virus-free negative control. The polyomovirus BK PCR mixture in a total volume of 50 μL contained 5 μL of 10X PCR buffer, 1.5 μL MgCl_2 _(50 mM), 1 μL dNTP (10 mM), 1 μL of each primer (20 pmol), 0.5 μL Taq (2.5 unit), and 5 μL of the sample DNA. The theromocycling condition of the PCR included one cycle at 94 °C for 3 min, 35 cycles (at 93 °C for 1 min, at 57 °C for 1 min, and at 72 °C for 1 min), ), and finally one cycle at 72°C for 5 min.

**Parvovirus B19:** The genomic DNA of parvovirus B19 was diagnosed in studied patients by specific original designed primers amplifying a fragment of *B19* gene using in an in-house nested-PCR protocol (Table 1). The sensitivity and specificity of this protocol was evaluated and optimized using confirmed parvovirus B19 genomic DNA as positive control and a laboratory-documented virus-free negative control. The ingredients and thermocycling conditions of simple and nested steps of PCR protocol were the same. The PCR mixture in a total volume of 50 μL contained 5 μL of 10X PCR buffer, 1.5 μL of MgCl_2_ (50 mM), 1 μL dNTP (10 mM), 1 μL of each primer (40 pmol), 0.5 μL Taq (2.5 unit), and 5 μL of the sample DNA. The theromocycling condition included one cycle at 94 °C for 1 min , 25 cycles (at 94 °C for 30 sec, at 50 °C for 30 sec, and at 72 °C for 30 sec), and finally, one cycle at 72 °C for 5 min. 

**Hepatitis C and G viruses:** The presence of genomic RNA of hepatitis C and G viruses was analyzed in studied patients using an in-house multiplex nested-RT-PCR. The cDNA was synthesized from genomic RNA of hepatitis C and G viruses in cDNA mix with a total volume 20 μL including 0.2 mM of dNTP, 0.01 mg/mL of random hexamer, 7.5 U/mL of Moloney murine leukemia virus reverse transcriptase (M-Mulv-RT), 1 U/mL of ribonuclease inhibitor, and 4 μL of 5X reverse transcriptase buffer, on 3 μL of extracted RNA. The thermocycling condition included one cycle at 25 °C for 60 min and one cycle at 72 °C for 10 min. The simple and nested steps of multiplex PCR were run using specific primer pairs that designed to amplify the 5′ untranslated regions of hepatitis C and G viruses (Table 1).

In the simple multiplex PCR step, the PCR mixture contained 2 μL of cDNA, 0.1 pmol/μL of primers, 0.2 mMol of dNTPs, 2.5 U of Taq DNA polymerase, 2.5 μL of 10X PCR buffer, and 1.5 mMol of MgCl_2_; 2 μL of simple PCR product was used in nested PCR step with the same PCR mix condition. The thermocycling condition for simple PCR step was initiated with one cycle at 95 °C for 5 min, 25 cycles (at 94 °C for 50 sec, at 55 °C for 40 sec, and at 72 °C for 50 sec), and finalized with one cycle at 72 °C for 3 min. The thermocycling condition of nested PCR step was initiated with one cycle at 95 °C for 5 min, 35 cycles (at 94 °C for 40 sec, at 64 °C for 35 sec, and at 72 °C for 40 sec), and finally, one cycle at 72 °C for 3 min.

## Results

The mean age of patients was 33 (range: 15–67) years. Of 27 patients, 16 (59%) were male. Table 2 shows the clinical presentation of the patients. History of viral infection is presented in Tables 3.

**Table 2:** Clinical presentation of the studied patients

Hematology Diagnosis	Symptoms	Final Disease	Sex	Age	Patients No.
Thrombocytopenia, Hemoglobinopathy	Petechia	Acute myelogenous leukemia	F	33	1
Thrombocytopenia, Hemoglobinopathy	Petechia	Idiopathic thrombocytopenic purpura, Acute myelogenous leukemia	F	29	2
Pancytopenia	Fever, petechia,	Transient bone marrow suppression	M	39	3
Pancytopenia	Fever, chills, body pain	Transient bone marrow suppression	M	58	4
Pancytopenia	Fever, body pain	Transient bone marrow suppression	F	20	5
Pancytopenia	Fever, sleeping disorder	Myelodysplastic syndrome	M	57	6
Pancytopenia	Fever, abdominal pain, diarrhea	Transient bone marrow suppression	M	22	7
Pancytopenia	Fever, malaise, body pain	Transient bone marrow suppression	F	24	8
Pancytopenia	Fever, malaise, chills	Transient bone marrow suppression	F	38	9
Pancytopenia	Fever, chills, vomiting	Acute lymphoblastic leukemia	F	16	10
Thrombocytopenia	GVHD, diarrhea, vomiting	Acute lymphoblastic leukemia, Bone marrow transplantation	M	37	11
Pancytopenia	Body pain	Transient bone marrow suppression	M	67	12
Pancytopenia	Weakness, body pain	Transient bone marrow suppression	M	25	13
Pancytopenia	Fever, chills	Transient bone marrow suppression	M	55	14
Pancytopenia	Fever, chills	Transient bone marrow suppression	F	54	15
Pancytopenia	Fever, petechia, vomiting	Acute myelogenous leukemia	F	15	16
Pancytopenia	Fever, diarrhea	Transient bone marrow suppression	M	32	17
Pancytopenia	Fever, malaise, chills	Acute lymphoblastic leukemia	M	33	18
Pancytopenia	Fever, diarrhea	Transient bone marrow suppression	F	48	19
Pancytopenia	Fever, chills, petechia	Transient bone marrow suppression	M	22	20
Pancytopenia	Fever, chills	Transient bone marrow suppression	M	19	21
Pancytopenia	Fever, chills	Myelodysplastic syndrome	M	35	22
Pancytopenia	Fever, chills	Idiopathic thrombocytopenic purpura	M	41	23
Pancytopenia	Fever, chills	Transient bone marrow suppression	F	24	24
Pancytopenia	Fever, chills	Transient bone marrow suppression	F	26	25
Leukopenia	Fever, chills	Myelodysplastic syndrome	M	38	26
Pancytopenia	Fever, chills	Transient bone marrow suppression	M	15	27

**Table 3:** Viral infections in patients with transient bone marrow suppression

Patients No.	BKV	HGV	HCV	HBV.B	HBV.P	ADV
1	–	–	–	–	+	–
2	–	–	–	–	–	–
3	–	–	–	–	–	–
4	–	–	–	–	–	–
5	–	–	–	–	+	–
6	–	–	–	+	–	–
7	–	–	–	–	–	–
8	–	–	–	+	+	–
9	–	+	–	–	+	–
10	–	–	–	–	+	–
11	–	–	–	–	–	–
12	–	–	–	+	+	–
13	–	+	+	+	–	–
14	–	–	–	+	–	–
15	–	–	–	–	–	–
16	–	–	–	–	–	–
17	–	–	–	+	–	–
18	–	–	–	–	–	–
19	–	–	–	–	+	–
20	–	–	–	–	+	–
21	–	–	–	–	–	–
22	–	–	–	+	–	+
23	–	–	–	–	–	+
24	–	–	+	+	–	–
25	–	–	–	–	–	–
26	–	–	–	–	–	–
27	–	+	+	–	+	–

BKV: Polyomavirus BK; HGV: Hepatitis G virus; HCV: Hepatitis C virus; HBV.B: Hepatitis B virus in blood; HBV.P: Hepatitis B virus in plasma; ADV: Adenovirus

**Molecular prevalence of DNA viruses**

Parvovirus B19 was found in 7 (26%) and the DNA of hepatitis B virus was detected in 10 (37%) of 27 studied patients. Viremia of adenovirus infection was diagnosed in 2 (7%) of 27 patients. However, we could not detect genomic DNA of polyomovirus BK in plasma samples of the studied patients (Table 3).

**Molecular prevalence of RNA viruses**

The viremia of hepatitis C virus was found in 3 (11%) of 24 studied patients. The genomic RNA of hepatitis G virus was also found in 3 (11%) of 24 patients (Table 3).

**Prevalence of viral coinfections**

Some of the patients were infected with more than one virus. Coinfection by two viruses was found in a few studied patients: hepatitis B and G in two; hepatitis B and C in one; and hepatitis C and G in another two patients. Triple infection of hepatitis B, C and G viruses was simultaneously found in only one patient (Table 1).

**Final clinical outcomes**

The final laboratory and clinical condition of studied patients are presented in Tables 2. In 3 of 27 patients, acute myelogenous leukemia was finally confirmed (Tables 2). Acute lymphoblastic leukemia was also confirmed in 3 of 27 patients (Tables 2). Myelodysplastic syndrome was the final diagnosis in other three patients. Idiopathic thrombocytopenic purpura was documented in two of the studied patients (Tables 2). 

## Discussion

Based on several earlier clinical and laboratory findings, the association of a number of viral infections with pathophsiology of transient bone marrow suppression is suggested [18]. Therefore, in this study the prevalence of some DNA and RNA viral infections were evaluated in patients with transient bone marrow suppression. Parvovirus B19 is one of the most prevalent viral infections which may have etiological role in transient bone marrow suppression [19]. The parvovirus B19 infection may lead to transient anemia and reticulocytopenia, as well as neutropenia and thrombocytopenia in hematological healthy population [17, 20, 21]. Earlier studies present a strong association (86%) between transient aplastic crisis with recent parvovirus B19 infections [22]. Some cases with chronic neutropenia have also been related to parvovirus B19 infection [22]. In keeping with these earlier reports, diagnosis of the high prevalence of 26% of parvovirus B19 infection in studied patients may emphasize the etiologic role of this viral infection in pathogenesis of transient bone marrow suppression. However, the less common association between transient pancytopenia and parvovirus B19 infection was reported in other studies [17, 23, 24].

Blood-borne hepatitis viruses with their extrahepatic pathogenesis may have role in the genesis of transient bone marrow suppression. Hepatitis B virus can infect virtually all cellular components of the hematopoietic system including the peripheral blood leukocytes and bone marrow cells [25]. The sporadic cases were reported with hepatitis B virus infection that has been shown bone marrow suppression. Hepatitis B virus infection was also confirmed in progenitor bone marrow cells of these patients [25]. In others studies, hepatitis B virus DNA sequences were identified in the peripheral blood cells of patients with hepatitis B virus infection [25, 26]. Basically, hepatitis B virus suppresses the development of colony-forming cells of both myelomonocytic and lymphoid lineages [25]. Based on extrahepatic potential of hepatitis B virus infection in this study, the high prevalence of hepatitis B virus infection was also found in 37% of the studied patients. This high frequency represented a possible etiologic role of hepatitis B virus in the genesis of transient bone marrow suppression—a hypothesis that needs to be further studied. 

Hepatitis C virus is a hepatotropic virus with a number of associated extrahepatic manifestations including hematological disorders. Hepatitis C virus infection is similar to hepatitis B virus; recently it was found to be associate with bone marrow suppression [26]. The prevalence of hepatitis C virus-related thrombocytopenia increased significantly (5–9 folds) along with liver disease severity and 20.3% of patients with chronic viral hepatitis C also had thrombocytopenia [25, 27, 28]. Hepatitis C virus infection is highly associated with thrombopoietin levels, platelet counts, liver function impairment, and the severity of hepatic fibrosis [29, 30]. Other studies have shown increasing thrombopoietin levels and platelet counts after interferon therapy of patients with hepatitis C viral infection [31]. In another study, the likelihood of thrombocytopenia in older patients infected with hepatitis C virus was three times that of patients in other age groups [26]. In this study, hepatitis C virus RNAmia was found in 11% of the studied patients. Like hepatitis C virus, hepatitis G virus may have lymphotropic effects; the role of hepatitis G virus in the pathogenesis of bone marrow suppression is not well-understood yet. In an earlier study, hepatitis G virus RNA was found in serum samples of five patients with aplastic anemia. Hepatitis G viral genome was also found in bone marrow of 19% of the patients [32]. In another study, the genomic RNA of hepatitis G virus was found in a patient with aplastic anemia in whom other risk factors were ruled out [33]. Nonetheless, in another study, none of the bone marrow transplant patients with aplastic anemia was found positive for hepatitis G virus. In our study, the prevalence of hepatitis G and hepatitis C virus infection was similar. The role of hepatitis G in pathogenesis of bone marrow depression needs further studies.
